# Dynamic Metabolic Zonation of the Hepatic Glucose Metabolism Is Accomplished by Sinusoidal Plasma Gradients of Nutrients and Hormones

**DOI:** 10.3389/fphys.2018.01786

**Published:** 2018-12-12

**Authors:** Nikolaus Berndt, Hermann-Georg Holzhütter

**Affiliations:** ^1^Computational Biochemistry Group, Institute of Biochemistry, Charite—University Medicine Berlin, Berlin, Germany; ^2^Institute for Computational and Imaging Science in Cardiovascular Medicine, Charite—University Medicine Berlin, Berlin, Germany

**Keywords:** metabolism, metabolic zonation, kinetic model, multiscale model, gene expression

## Abstract

Being the central metabolic organ of vertebrates, the liver possesses the largest repertoire of metabolic enzymes among all tissues and organs. Almost all metabolic pathways are resident in the parenchymal cell, hepatocyte, but the pathway capacities may largely differ depending on the localization of hepatocytes within the liver acinus-a phenomenon that is commonly referred to as metabolic zonation. Metabolic zonation is rather dynamic since gene expression patterns of metabolic enzymes may change in response to nutrition, drugs, hormones and pathological states of the liver (e.g., fibrosis and inflammation). This fact has to be ultimately taken into account in mathematical models aiming at the prediction of metabolic liver functions in different physiological and pathological settings. Here we present a spatially resolved kinetic tissue model of hepatic glucose metabolism which includes zone-specific temporal changes of enzyme abundances which are driven by concentration gradients of nutrients, hormones and oxygen along the hepatic sinusoids. As key modulators of enzyme expression we included oxygen, glucose and the hormones insulin and glucagon which also control enzyme activities by cAMP-dependent reversible phosphorylation. Starting with an initially non-zonated model using plasma profiles under fed, fasted and diabetic conditions, zonal patterns of glycolytic and gluconeogenetic enzymes as well as glucose uptake and release rates are created as an emergent property. We show that mechanisms controlling the adaptation of enzyme abundances to varying external conditions necessarily lead to the zonation of hepatic carbohydrate metabolism. To the best of our knowledge, this is the first kinetic tissue model which takes into account in a semi-mechanistic way all relevant levels of enzyme regulation.

## Introduction

The tightly controlled switch between hepatic uptake and release of glucose keeps the plasma glucose concentrations within a range between 4 and 10 mM despite largely varying carbohydrate intake and utilization. This homeostatic function of the liver with respect to plasma glucose is achieved by several enzyme-regulatory mechanisms acting on different time scales. On the short term, hormone-dependent reversible enzyme phosphorylation and changes of reaction rates elicited by concentration changes of reaction substrates/products and allosteric modulators enable a metabolic response within seconds or minutes. Recurrent activation of these fast regulatory modes is typically accompanied by slow changes in the abundance of metabolic enzymes on a time scale of hours to days (Hopgood et al., 1973; Weinberg and Utter, 1980). Both the fast and slow mode of enzyme regulation are important for the regulation of the glucose exchange flux between hepatocytes and blood plasma (Bulik et al., 2016), Owing to concentration gradients of oxygen, metabolites, hormones, and morphogens along the hepatic capillaries (sinusoids) the expression of metabolic enzymes may differ in various zones of the liver acinus. For example, the oxygen pressure decreases by 50% along the porto-central axis of the acinus (Jungermann and Kietzmann, 2000). This goes in line with the number and structure of mitochondria (Schmucker et al., 1978) and glycolytic capacities in the periportal and pericentral zone (Braeuning et al., 2006). Hepatocytes close to the portal pole (zone 1) experiencing the highest concentration of oxygen pressure are predestined for strong ATP-demanding anabolic pathways like gluconeogenesis and urea synthesis. Contrary, hepatocytes close to the venous pole of the acinus (zone 3) experience the lowest oxygen concentrations and thus possess a high glycolytic capacity, a typical feature of cells working under conditions of permanent oxygen deprivation. The heterogeneous allocation of gluconeogenetic and glycolytic capacities to different hepatocytes along the porto-central axis may even result in a situation where a certain fraction of glucose produced by periportal cells is used to fuel the glycolysis of pericentral cells (Berndt et al., 2018).

Several blood-born factors have been identified as regulators of zone-dependent gene expression of metabolic enzymes. Oxygen, glucose, the hormones glucagon and insulin, the morphogens Wnt and hedgehog and the growth factor HGF belong to the best studied factors. The various factors appear to act in a hierarchical fashion whereby the gradients of morphogens and growth factors create a basic expression pattern that is further modulated by nutrition-related factors such as oxygen, glucose, fatty acids and the hormones insulin and glucagon. In this work, we will focus on the latter group of modifiers, i.e., we restrict our model to the metabolic response of the liver to nutritional challenges and oxygen availability.

Metabolic adaptation of hepatocytes to varying oxygen pressures is mainly controlled by hypoxia-inducible transcription factors (HIFs), heterodimeric complexes consisting of a constitutively expressed β-subunit and an oxygen-sensitive α-subunit. In the liver, HIF-1α regulates primarily glycolytic genes whereas HIF-2α is known to primarily regulate genes involved in cell proliferation and iron metabolism (Ramakrishnan and Shah, 2017). In line with the falling oxygen pressure along the porto-central axis, HIFαs were found with higher levels in the less aerobic pericentral zone (Kietzmann et al., 2001). Besides oxygen, the pancreatic hormones insulin and glucagon are important drivers of zone-dependent differences in enzyme activities. The regulatory role of these hormones is 2-fold. They control the cellular cAMP level in an antagonistic manner and thus exert opposite effects on the reversible phosphorylation of key regulatory enzymes of glycolysis and gluconeogenesis as PFK2, PK, and PEPCK. The hepatic clearance of the two hormones by endocytic uptake into hepatocytes creates a concentration gradient along the porto-central axis which entails zone-dependent differences in the phosphorylation level of interconvertible enzymes. With respect to gene expression of metabolic enzymes, insulin and glucagon also control the efficiency of several transcription factors as ChREBP, SREBP-1c, CREB, and Foxo (Han et al., 2016). Both actions of glucagon and insulin are tightly interrelated and function in part through the same mechanisms. For example, the cAMP-activated protein kinase A (PKA) is responsible for phosphorylation of interconvertible enzymes such as FBPFK2 and PK, as well as for the phosphorylation of the transcription factors ChREBP and CREP (Uyeda and Repa, 2006). cAMP is produced by glucagon-induced activation of the adenylate cyclase and degraded by insulin-stimulated cAMP phosphodiesterase. Consequently, the protein level of key regulatory enzymes reflects the integral hormone levels over longer time periods.

In this work we included dynamic changes in the abundance of metabolic enzymes into our previously developed multi-scale tissue model of hepatic glucose metabolism (Berndt et al., 2018). The rates of protein synthesis and degradation were modeled by phenomenological rate equations which were parameterized by using experimentally determined protein levels at varying concentrations of oxygen, glucose, insulin, and glucagon. The central aims of our work were (i) to provide a proof of principle for integrating in a self-consistent manner the temporal gene expression of enzymes into kinetic models of cellular metabolism, (ii) to lend further support to the concept of post-differentiation patterning according to which metabolic zonation is driven by gradients of oxygen, nutrients and hormones in the capillary blood and (iii) to present a modeling approach that obviates the requirement to measure the cellular abundance of metabolic enzymes (e.g., by quantitative proteomics) in different physical states of the liver, a procedure burdened with many problems as, for example, invasive tissue sampling and protein quantification in cells separated from different zones.

## Model Description

The model combines a mathematical model of the sinusoidal tissue unit (STU) (Berndt et al., 2018) with a kinetic model of the protein turnover of key regulatory enzymes.

### Compartment Model of Metabolite and Hormone Transport in the Sinusoidal Tissue Unit (STU)

**Figure 1:**
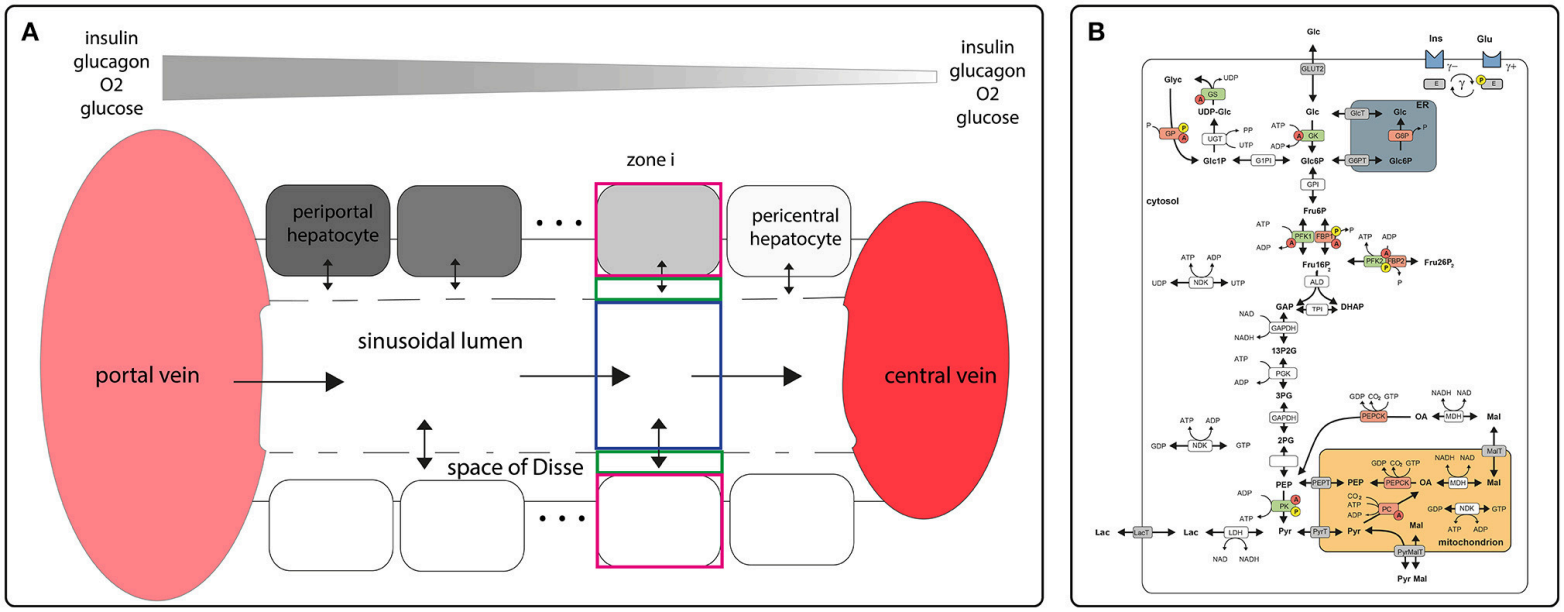
Schematic model representation **(A)** sinusoidal blood flow model describing blood flow, nutrient and hormone distribution within the sinusoids. The model encompasses a single sinusoid, the adjacent space of Disse and the surrounding layer of hepatocytes. It is described by morphological parameters (blood vessel radius, thickness of the space of Disse, hepatocyte thickness, hepatocyte number, sinusoid length, degree of fenestration), and systemic parameters (central and portal vein hydrostatic pressure, plasma and lymph oncotic pressure, diffusion coefficients); **(B)** model of carbohydrate metabolism encompassing glycolysis, glyconeogenesis and glycogen synthesis and utilization. These pathways comprise the enzymes Glucokinase (GK), Glucose-6-phosphate isomerase (GPI), Phosphofructokinase 1 (PFK1), Aldolase (ALD), Triosephosphate isomerase (TPI), Glyceraldehydephosphate dehydrogenase (GAPDH), Phosphoglycerate kinase (PGK), Phosphoglycerate mutase (PGM), Enolase (EN), Pyruvate kinase (PK) Lactate dehydrogenase (LDH), Glucose-6-phosphate phosphatase (G6P), Phosphofructokinase 2 (PFK2), Fructose-2,6-bisphosphatase (FBP2), Fructose-1,6-bisphosphatase (FBP1), Phosphoenolpyruvate carboxykinase (PEPCK), Pyruvate carboxylase (PC), Nucleoside-diphosphate kinase (NDK), Malate dehydrogenase (MDH), Pyrophosphatase (PPASE), Glucose-1-phosphate isomerase (P1PI), Glycuronosyltransferase (UGT), Glycogen phosphorylase (GP), Glycogen synthase (GS) and transporters (ER <-> cytosol: Glucose-6-phosphate transporter (Glc6PT), Glucose transporter (GlcT); mitochondrion <-> cytosol: Pyruvate transporter (PYRT), Phosphoenolpyruvate transporter (PEPT), Malate transporter (MALT); extern <-> cytosol: Glucose transporter 2 (GLUT2), Lactate transporter (LACT). Enzymes that are phosphorylated or dephosphorylated in response to insulin (Ins) and glucagon (Glu) stimulus are marked by a yellow P, allosteric modification of enzymes is marked by a red A. The model contains the metabolites: glucose (Glc), glucose-6-phosphate (Glc6P), fructose-6-phosphate (Fru6P), fructose-1,6-bisphosphate (Fru16P_2_), glyceraldehydephosphate (GraP), dihydroxyacetonephosphate (DHAP), 1,3-bisphosphoglycerate (13P2G), 3-phosphoglycerate (3PG), 2-phosphoglycerate (2PG), phosphoenolpyruvate (PEP), pyruvate (Pyr), lactate (Lac), malate (Mal), oxaloacetate (OA), glucose-1-phosphate (Glc1P), UDP-glucose (UDP-glc), glycogen, fructose-2,6-bisphosphate (Fru26P_2_). The cofactors NADH, NAD, ATP, ADP, UTP and UDP are not treated as dynamic variables. All physiological metabolites produced or consumed in the hepatocyte during glycolysis and gluconeogenesis are comprised into lactate. Figures adapted from Berndt et al. (2018).

Structurally, the STU is defined by a single sinusoid, the adjacent space of Disse and a monolayer of hepatocytes flanking the space of Disse (see Figure 1A). Functionally, the model describes the exchange of oxygen, metabolites and hormones between the sinusoidal blood, the space of Disse and the hepatocytes and the glucose metabolism within hepatocytes. The transport of metabolite and hormones within the STU is driven by diffusion and directional transport along the flow of water and blood. Lateral blood flow in the vessel is described by Hagen-Poiseuille law for fluid flow through a cylinder, water flow in the space of Disse is described by Hagen-Poiseuille law for fluid flow in a hollow cylinder. Exchange of water between the vessel and the space of Disse is driven by hydrostatic and oncotic pressure difference between the blood vessel and the space of Disse.

Conceptually, the STU was divided into N_H_ zones, where N_H_ is the number of hepatocytes along the porto-central axis. Each zone is made up of the sinusoid volume, the space of Disse and the hepatocyte (Figure 1A). Within one zone, the concentration of metabolites and hormones is given by a single value. The mathematical description of the STU model and a complete list of parameters used can be found in Berndt et al. (2018).

### Kinetic Model of Hepatocyte Glucose Metabolism

The reaction scheme for the glucose metabolism of a single hepatocyte is depicted in Figure 1B. It consists of the pathways for glycolysis, glyconeogenesis, glycogen synthesis and degradation. The time-dependent variation of metabolite concentrations is given by first-order differential equations. The liver specific enzymatic rate laws take into account substrate regulation, allosteric regulation and hormonal regulation by hormone-dependent reversible phosphorylation (Bulik et al., 2016).

### Kinetic Model of Hormonal Signaling

The pancreatic hormones glucagon and insulin are released into the portal vein in response to the plasma glucose concentration and are partially cleared during their passage through the liver. Hence, there is a difference between their plasma concentrations determined in peripheral blood samples and effective intra-hepatic concentrations. This difference was taken into account by setting the concentration values of insulin and glucagon in the periportal blood to the 2-fold of their plasma values (Balks and Jungermann, 1984). The rate of intra-hepatic hormone clearance via receptor binding and subsequent endocytosis was put proportional to the binding and signaling strength of the hormone. We used empirical transfer functions to describe the relationship between glucose and hormone concentrations in the plasma and the relationship between and the phosphorylation state of interconvertible enzymes as described in Bulik et al. (2016). A detailed description of the functions and their construction can be found in Berndt et al. (2018).

### Kinetic Model of Protein Turnover

The temporal change of the protein level P^ENZ^ of a metabolic enzyme (ENZ) is given by the difference between the rates of protein synthesis ${\text{v}}_{\text{syn}}^{\text{ENZ}}\left(\text{E}\right)$ and protein degradation ${\text{v}}_{\text{deg}}^{\text{ENZ}}\left(\text{E}\right)$:

(1) $$\begin{array}{r} \frac{\text{d}}{\text{dt}}{\text{P}}^{\text{ENZ}}={\text{v}}^{\text{ENZ}}\left(\text{E}\right)={\text{v}}_{\text{syn}}^{\text{ENZ}}\left(\text{E}\right)-{\text{v}}_{\text{deg}}^{\text{ENZ}}\left(\text{E}\right) \end{array}$$

The right-hand side of equation (1), *v*^ENZ^(E), represents the turnover rate of the enzyme protein. It is controlled by modulators affecting either the synthesis or the degradation or both. Note that the enzyme level P^ENZ^ scales linearly with the maximal rate of the enzyme. The rate equations of protein synthesis and degradation both depend on the momentary concentration of at least one of the four modulators E_i_, *i* = 1 (insulin), *i* = 2 (glucagon), *i* = 3 (glucose), *i* = 4 (oxygen) considered in the model. The general structure of the rate equation for the protein synthesis of enzyme ENZ reads.

(2) $$\begin{array}{r} {\text{v}}_{\text{syn}}^{\text{ENZ}}={\text{k}}_{\text{syn}}^{\text{ENZ}}\overset{4}{\underset{i=1}{\prod }}\left({\text{k}}_{i}^{\text{ENZ}}\pm {\text{f}}_{i}^{\text{ENZ}}\right) \end{array}$$

where $k_{i}^{\text{ENZ}}$ is a constant determining the basal synthesis rate and f_i_ is a nonlinear function of the i-th modulator. The “+” sign holds if E_i_ is an activator (inductor) of protein synthesis, the (–) sign holds If E_i_ is an inhibitor (repressor). If E_i_ has not been reported so far to exert an effect on the protein synthesis of enzyme ENZ it holds ${\text{k}}_{\text{i}}^{\text{ENZ}}=1$ and ${\text{f}}_{\text{i}}^{\text{ENZ}}=0$ Numerical values for the rate constants $k_{syn}^{enz}$ and $k_{\deg}^{enz}$ were fixed in such a manner that for a normal 24 h plasma profile (see below) the zone- and time averaged protein levels coincided with the stationary protein levels as reported in Bulik et al. (2016). Numerical values of all other kinetic parameters were obtained by adjusting the rate equations to experimentally determined protein levels at varying concentrations of the four possible modulators (see Supplement 1). Table 1 depicts the rate equations for the synthesis and degradation of those enzymes of hepatic glucose metabolism possessing in the model timely variable protein levels.

**Table 1:** Synthesis and degradation rates of the regulatory enzymes of hepatic carbohydrate.

**Enzyme name**	**$v_{syn}^{ENZ}$**	**$v_{deg}^{ENZ}$**	**References**
Glucose transporter	$k_{syn}^{glcT}\cdot \left(k_{1}+k_{2}\frac{glc_{ext}}{glc_{ext}+K_{glc_{ext}}}\right)$ $k_{syn}^{glcT}=6.1\cdot 10^{-3}$ *k*_1_ = 0.8 *k*_2_ = 6 *K*_*gl*_*c*__*ext*__ = 15 *mM*	$k_{\deg}^{glcT}$$k_{\deg}^{glcT}=\;\frac{\ln\left(2\right)}{40}$	Postic et al., 1993; Weinstein et al., 1994
Glucokinase	$k_{syn}^{gk}\cdot \left(k_{1}-k_{2}\frac{o_{2}^{n}}{o_{2}^{n}+K_{o_{2}}^{n}}\right)\cdot \left(k_{3}\frac{ins}{ins+K_{ins}}\right)$ $k_{syn}^{glcT}=6.5\cdot 10^{-3}$ *k*_1_ = 2 *k*_2_ = 1 *K*_*o*_2__ = 80 *mmHG n* = 15 *k*_3_ = 10 *K*_*ins*_ = 500 *pM*	$k_{\deg}^{gk}$$k_{\deg}^{gk}=\;\frac{\ln\left(2\right)}{15}$	Dice and Goldberg, 1975; Sibrowski et al., 1981, 1982; Iynedjian et al., 1989; Kietzmann et al., 1997
Glucose-6-phosphatase	$k_{syn}^{g6p}\cdot \left(k_{1}-k_{2}\frac{glucagon^{n1}}{glucagon^{n1}+K_{glucagon}^{n}}\right)\cdot \left(k_{3}+k_{4}\cdot k_{eff\;}\frac{glc_{ext}^{n2}}{glc_{ext}^{n2}+K_{glc_{ext}}^{n2}}\right)$ $k_{syn}^{g6p}=1.16\cdot 10^{-2}$ *k*_1_ = 1 *k*_2_ = 0.8 *n*1 = 3 *K*_*glucagon*_ = 100 *pM k*_3_ = 1 *k*_4_ = 15 *k*_*eff*_ = 0.3 *K*_*gl*_*c*__*ext*__ = 17 *mM n*2 = 20	$k_{\deg}^{g6p}$$k_{\deg}^{g6p}=\;\frac{\ln\left(2\right)}{48}$	Leskes et al., 1971; Argaud et al., 1996; Massillon, 2001
Phosphofructokinase 1	$k_{syn}^{pfk1}$ $k_{syn}^{pfk1}=7.8\cdot 10^{-3}$	$k_{\deg}^{pfk1}$$k_{\deg}^{pfk1}=\;\frac{\ln\left(2\right)}{22+k_{2}\cdot \frac{ins^{n}}{ins^{n}+K_{ins}^{n}}}$*k*_2_ = 80 *hK*_*ins*_ = 100 *pMn* = 1.5	Dunaway and Weber, 1974; Dunaway et al., 1978
Fructosebis-phosphatase 1	$k_{syn}^{fbp1}\cdot \left(k_{1}+k_{2}\cdot \frac{glu^{n}}{glu^{n}+K_{glu}^{n}}\right)$ $k_{syn}^{fbp1}=2.99\cdot 10^{-2}$ *k*_1_ = 0.25 *k*_2_ = 1 *K*_*glu*_ = 100 *pM n* = 3	$k_{\deg}^{fbp1}$$k_{\deg}^{g6p}=\;\frac{\ln\left(2\right)}{40}$	Dice and Goldberg, 1975; Zalitis and Pitot, 1979; el-Maghrabi et al., 1991
Phosphofructo-kinase 2/Fructosebis-phosphatase 2	$k_{syn}^{pk}\cdot \left(k_{1}-k_{2}\cdot \frac{o_{2}^{n}}{o_{2}^{n}+K_{o_{2}}^{n}}\right)\cdot \left(k_{3}-k_{4}\frac{glu}{glu+K_{glu}}\right)$ $k_{syn}^{pfk2}=1.1\cdot 10^{-2}$ *k*_1_ = 2 *k*_2_ = 1 *K*_*o*_2__ = 75 *mmHG n* = 10 *k*_3_ = 1 *k*_4_ = 1 *K*_*glu*_ = 80 *pM*	$k_{\deg}^{pfk2}$$k_{\deg}^{g6p}=\;\frac{\ln\left(2\right)}{69}$	Dunaway and Weber, 1974; Dunaway et al., 1978; Rosa et al., 1993; Minchenko et al., 2003
Pyruvate kinase	$k_{syn}^{pk}\cdot \left(k_{1}-k_{2}\cdot \frac{o_{2}^{n}}{o_{2}^{n}+K_{o_{2}}^{n}}\right)\cdot \left(k_{3}-k_{4}\frac{glu}{glu+K_{glu}}\right)\cdot \left(k_{5}+k_{6}\frac{ins^{n2}}{ins^{n2}+K_{ins}^{n2}}\right)$ $k_{syn}^{pk}=1.06\cdot 10^{-2}$ *k*_1_ = 3.2 *k*_2_ = 1 *K*_*o*_2__ = 75 *mmHG n* = 10 *k*_3_ = 1 *k*_4_ = 0.5 *K*_*glu*_ = 150 *pM k*_5_ = 0.2 *k*_6_ = 1 *K*_*ins*_ = 500 *pM n*2 = 2	$k_{\deg}^{pk}$$k_{\deg}^{pk}=\;\frac{\ln\left(2\right)}{69}$	Hopkirk and Bloxham, 1979, 1980; Noguchi et al., 1985; Wölfle and Jungermann, 1985
Pyruvate carboxylase	$k_{syn}^{pc}\cdot \left(k_{1}+k_{2}\cdot \frac{glu}{glu+K_{glu}}\right)$ $k_{syn}^{pc}=1.47\cdot 10^{-2}$ *k*_1_ = 0.1 *k*_2_ = 1 *K*_*glu*_ = 150 *pM*	$k_{\deg}^{pc}$$k_{\deg}^{pc}=\;\frac{\ln\left(2\right)}{110}$	Weinberg and Utter, 1979, 1980
Phosphoenol-pyruvate Carboxykinase	$k_{syn}^{pepck}\cdot \left(k_{1}+k_{2}\cdot \frac{o_{2}^{n1}}{o_{2}^{n1}+K_{o_{2}}^{n1}}\right)\cdot \left(k_{3}+k_{4}\frac{glu^{n2}}{glu^{n2}+K_{glu}^{n2}}\right)\cdot \left(k_{5}-k_{6}\frac{ins}{ins+K_{ins}}\right)$ $k_{syn}^{pepck}=3.43\cdot 10^{-2}$ *k*_1_ = 0.5 *k*_2_ = 2 *K*_*o*_2__ = 75 *mmHG n*1 = 10 *k*_3_ = 1 *k*_4_ = 3 *n*2 = 0.5 *K*_*glu*_ = 0.2 *pM k*_5_ = 1 *k*_6_ = 0.8 *K*_*ins*_ = 100 *pM*	$k_{\deg}^{pepck}$$k_{\deg}^{pepck}=\;\frac{\ln\left(2\right)}{13}$	Nauck et al., 1981; Christ et al., 1988; Gabbay et al., 1996

In order to quantify the sensitivity of the turnover rate v^ENZ^(E) of a protein against small changes of an modulator E, we used the sensitivity (elasticity) coefficient as defined in metabolic control analysis:

(3) $$\begin{array}{r} {\text{SV}}^{\text{ENZ}}=\frac{\text{E}}{{\text{v}}^{\text{ENZ}}\left(\text{E}\right)}\frac{\partial v^{\text{ENZ}}\left(\text{E}\right)}{\partial \text{E}} \end{array}$$

**Figure 2:**
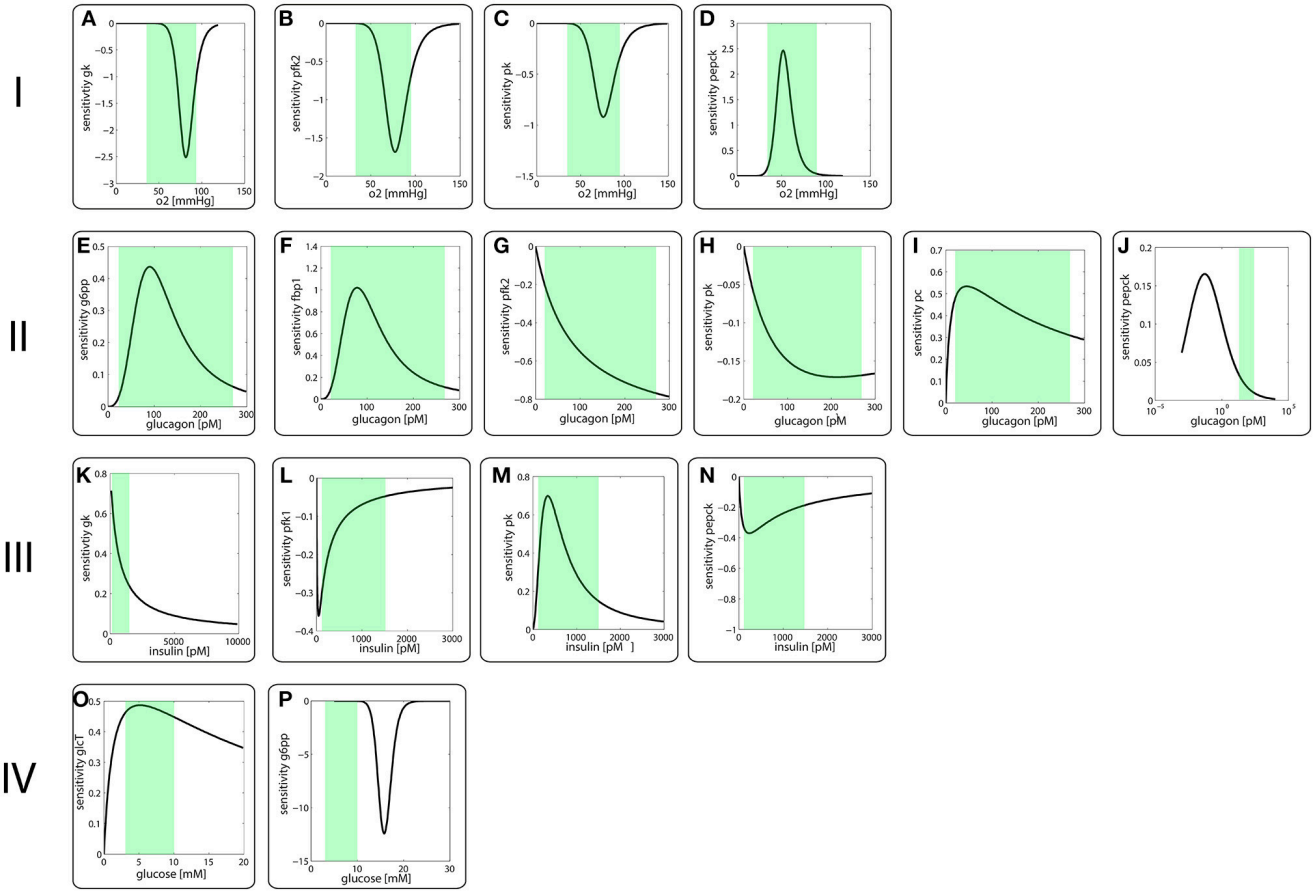
Sensitivity coefficients of protein turnover rates defined in equation (3) as function of modulator concentrations. I Sensitivity of protein turnover rates with respect to oxygen of **(A)** GK, **(B)** FBPFK2, **(C)** PK, **(D)** PEPCK II Sensitivity of protein turnover rates with respect to **(E)** G6PP, **(F)** FBP1, **(G)** FBPFK2, **(H)** PK, **(I)** PC, **(J)** PEPCK III Sensitivity of protein turnover rates with respect to **(K)** GK, **(L)** PFK1, **(M)** PK, **(N)** PEPCK IV Sensitivity of protein turnover rates with respect to **(O)** GlcT, **(P)** G6PP The green-shades areas indicate the reported physiological concentration range of the respective modulator.

Figure 2 depicts the sensitivity coefficients for the turnover rates of the nine enzyme proteins with variable expression level as function of the four modulators oxygen (I), glucagon (II), insulin (III) and glucose (IV). Except for the sensitivities of the PEPCK and G6PP turnover with respect to glucagon and glucose, respectively, the extremum of all other sensitivity characteristics lies within the reported physiological range of the related modulators (green-shaded areas in Figure 2). The sensitivity of G6PP turnover with respect to glucose becomes important in the diabetic case, where glucose levels can exceed 20 mM (see below).

## Results

### Dynamic Metabolic Zonation in a Well-Fed State of the Rat

First, we used the model to simulate the temporal variation of enzyme abundances, metabolite concentrations and fluxes within the various zones along the porto-central axis of the STU. The simulation was initiated with identical abundance of enzymes along the sinusoid which we set to the stationary mean protein abundance used in Bulik et al. (2016). We used as model input the diurnal glucose profile reported for the healthy normal liver of a fed rat (La Fleur et al., 1999) and carried out the numerical integration of the model over several 24 h cycles until there was no change in the 24 h enzyme and metabolite profiles.

**Figure 3:**
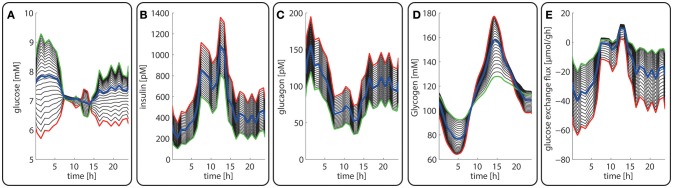
Diurnal variations in the plasma levels of glucose **(A)**, insulin **(B)**, glucagon **(C)**, cellular glycogen **(D)** and the glucose exchange flux **(E)** in different zones along the porto-central axis. The different curves refer to different spatial positions of hepatocytes, counted from periportal (red curve) to percentral (green curve). The bold blue line refers to the means values of the shown variable. Note that the red curves (= most portal cell) for the hormones and glucose are identical with their plasma profiles.

**Figure 4:**
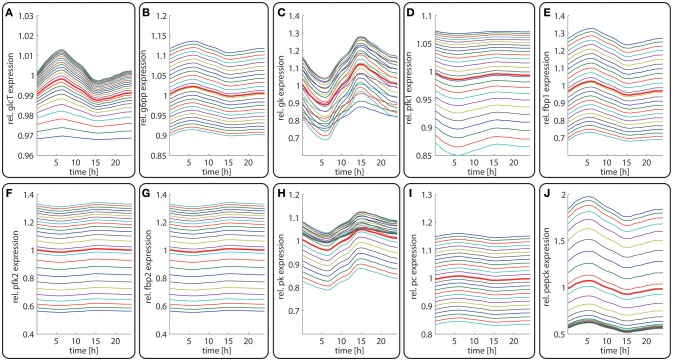
Diurnal variations in the relative abundance of glycolytic and gluconeogenetic enzymes within hepatocytes along the porto-central axis of a well-fed rat. The curves illustrate the relative deviation of protein abundances from the overall spatial and 24h- mean (= 24 h mean of the bold red curve). From the top to the bottom, the different curves refer to the spatial position of the hepatocyte counted from periportal to percentral. The bold red line refers to the average protein abundance across all cells. **(A)** GlcT, **(B)** G6PP, **(C)** GK, **(D)** PFK1, **(E)** FBPPFK1, **(F)** PFK2, **(G)** FBPPFK2, **(H)** PK, **(I)** PC, **(J)** PEPCK.

Even with identical enzyme abundances across all hepatocytes, there occurs a progressive decline of hormone plasma levels from the portal to the central pole due to the ongoing hormone uptake by hepatocytes in each zone. Moreover, oxygen uptake in one zone diminishes the available oxygen pressure seen by the cells in the adjacent zone toward the pericentral pole. As oxygen is not part of the model, we assumed a linear decrease in oxygen partial pressure from 90 mmHG in the periportal zone to 35 mm HG in the pericentral zone (Jungermann and Kietzmann, 2000; Allen et al., 2005). These initial gradients of hormones, glucose and oxygen feed back to the level of metabolic enzymes so that finally zone-dependent patterns of both enzyme abundances and metabolic variables (see Figures 3, 4) are generated.

Figures 3A–C depicts the timely variation of glucose, insulin, glucagon in various sinusoidal compartments. Intriguingly, the highest glucose concentrations in the very portal zone (see red curve in Figure 3A) are paralleled by the lowest glucose concentrations in the very central zone (green curve). This seemingly paradoxical situation is due to the fact that the high level of insulin and low level of glucagon strongly increase the glucose uptake capacity of hepatocytes such that the otherwise strong zone-dependent differences in the glucose exchange flux (see Figure 3E) almost disappear. The simulation also reveals large zone-dependent differences in the cellular dynamics of glycogen (Figure 3D). The variation of the glycogen content in portal cells is much more pronounced than in central cells.

The time-dependent variation in the protein levels of key glycolytic and gluconeogenetic enzymes in different zones are depicted in Figure 4. The uniform overall shape of the curves reflects essentially the daily variation of the plasma glucose level. Generally, the daily fluctuations of enzyme levels around their 24 h mean hardly exceed 10%. Thus, as long as the liver is repeatedly confronted with the same 24 h plasma profile of metabolites and hormones, timely variations of protein levels should have only a marginal impact on the hepatic control of the plasma glucose level.

**Figure 5:**
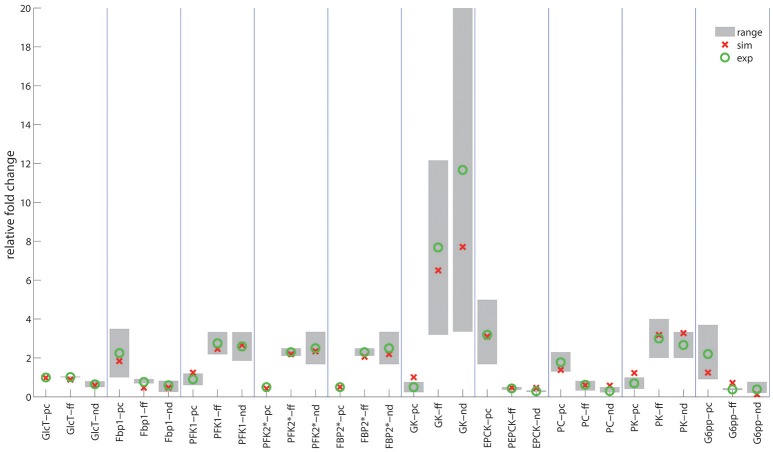
Ratio of enzyme levels in hepatocytes. Red circles indicate the computed ratio of protein levels. Green circles and gray bars indicate the mean value and the range of variability of various experimentally determined ratios. First columns (pc): Portal-to-central ratios (= 24h-averaged protein levels between hepatocyte #1 and hepatocyte # 25) for the well-fed state of the rat. Experimental data were taken from (Sharma et al., 1964; Katz et al., 1977a,b; Trus et al., 1980; Jungermann and Katz, 1982, 1989; Probst et al., 1982; Teutsch and Lowry, 1982; Miethke et al., 1985; Quistorff, 1985; Wölfle and Jungermann, 1985; Morselt et al., 1987; Chen and Katz, 1988; Wals et al., 1988; Evans et al., 1989; Frederiks et al., 1991; Jones and Titheradge, 1996; Minchenko et al., 2003). Second columns (ff): Fed-to-fasted ratios of 24h- and zone-averaged mean enzyme levels. Experimental data taken from (Dipietro and Weinhouse, 1960; Wimhurst and Manchester, 1970b; Ballard and Hopgood, 1973; Bock et al., 1973; Zalitis and Pitot, 1979; Cladaras and Cottam, 1980; Neely et al., 1981; Bahnak and Gold, 1982; Van Schaftingen and Hers, 1983; Donofrio et al., 1984; Colosia et al., 1988; Crepin et al., 1988; Thorens et al., 1990; Giffin et al., 1993; Gannon and Nuttall, 1997). Third columns (nd): Normal-to-diabetic ratios of 24h- and zone-averaged mean enzyme levels. Experimental data taken from Dipietro and Weinhouse, 1960; Salas et al., 1963; Exton and Park, 1965; Wimhurst and Manchester, 1970a; Chang and Schneider, 1971; Dunaway et al., 1978; Weinberg and Utter, 1980; Neely et al., 1981; Donofrio et al., 1984; Miethke et al., 1985; Colosia et al., 1988; Crepin et al., 1988; Thorens et al., 1990; Miralpeix et al., 1992; Slieker et al., 1992; Gannon and Nuttall, 1997; Raju et al., 1999; Manna and Jain, 2012.

In contrast to the modest time-dependent variations of protein levels, the computed zone-dependent differences of enzyme levels display a large scatter. The maximal differences between the enzyme endowment of hepatocytes closest to the portal and central pole lie between 0.1 [e.g., glucose transporter (glcT) and phosphofructokinase 1 (pfk1)] and 4.5 [phosphoenolpyruvate kinase (pepck)]. For the validation of these computational predictions, we calculated the 24 h-average protein levels of the first (most portal) and last (most central) hepatocyte and compared the ratio of the computed average protein levels with experimental data (see first columns for each enzyme in Figure 5). We further compared the ratio of 24 h- and zone-averaged mean protein levels between a fed and fasted rat and a diabetic and normal rat.

This analysis provided a good concordance between theoretical and experimental results. The only exception is the pyruvate carboxylase, a key regulatory enzyme of gluconeogenesis, were portal to central gradients could not be univocally explained by the reported oxygen dependency. Oxygen dependency accounts only for about 35% percent of the observed zonation (see Table 1 and Supplement 1).

### Dynamic Metabolic Zonation of the Liver During Adaptation to Fasting

**Figure 6:**
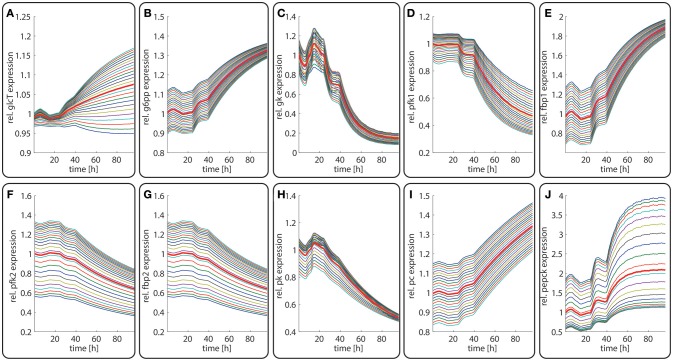
Diurnal variations in the relative abundance of glycolytic and gluconeogenetic enzymes within hepatocytes along the porto-central axis during the transition from a fed state (*t* = 0–24 h) to a fasted state (*t* > 24 h). The curves illustrate the relative deviation of protein abundances from the overall spatial and 24 h mean (= 24 h mean of the bold red curve). From the top to the bottom, the different curves refer to the spatial position of the hepatocyte counted from periportal to percentral. The bold red line refers to the average protein abundance across all cells. The vertical dotted line indicates onset of the starvation period. **(A)** GlcT, **(B)** G6PP, **(C)** GK, **(D)** PFK1, **(E)** FBPPFK1, **(F)** PFK2, **(G)** FBPPFK2, **(H)** PK, **(I)** PC, **(J)** PEPCK.

Next, we studied how the zonation of metabolic enzymes is affected if the liver has to cope with a fundamentally different nutritional regime. To this end, we simulated the zone-dependent dynamic changes of protein levels and metabolites during the transition from a fed state of the rat to a fasting state. The simulation started with the stable 24 h zonated enzyme profile that is established if the liver experiences recurrently the same plasma profile of a fed rat (see above). At time *t* = 24 h, the plasma profile of the fed rate was replaced by plasma profile of a fasted rat (La Fleur et al., 1999). The latter was only available for a time range of 24 h of fasting. After about 16 h of fasting, stable values of plasma metabolites were reached. Therefore, we used for time points *t* > 48 h (i.e., >24 h of fasting) for the model input a plasma profile that was composed of 6 repetitions of the last part (16–24 h) of the 24 h fasting plasma profile. As shown in Figure 6, the fed-to-fasting transition evokes a significant rise in the abundance of key gluconeogenetic enzymes (GlcT, FBP1, PC, PEPCK) and drop in the abundance of key glycolytic enzymes (GK, PFK1, PFK2, FBP2, PK) in all zones. For two enzymes, the GlcT and the PEPCK, the zone-dependent protein differences become more pronounced compared to the fed state. By contrast, for the GK, FBP1, GSPP, and PK the zone-dependent protein differences became smaller.

**Figure 7:**
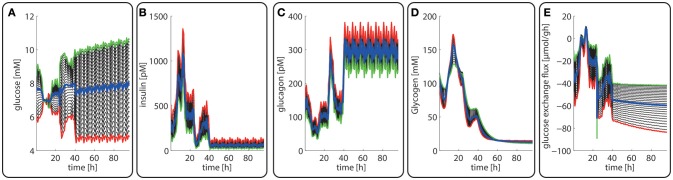
Diurnal variations in the plasma levels of glucose **(A)**, insulin **(B)**, glucagon **(C)**, cellular glycogen **(D)** and the glucose exchange flux **(E)** in different zones along the porto-central axis during the transition from a well-fed state (*t* = 0–24 h) to a fasted state (*t* > 24 h) of the rate. The different curves refer to different spatial positions of hepatocytes, counted from periportal (red curve) to percentral (green curve). The bold blue line refers to the means values of the shown variables. Note that the red curves (= most portal cell) for the hormones and glucose are identical with their plasma profiles.

The computed changes of enzyme profiles toward a more gluconeogenetic phenotype are accompanied by significant alterations of the intra-sinusoidal glucose gradient and the zone-dependent differences in the glucose exchange rate (Figure 7). Compared with the fed state, the porto-venous glucose difference becomes much larger in the fasted state (Figure 7A). The same holds for the glucose exchange rate (Figure 7D).

In agreement with experimental data (Babcock and Cardell, 1974), the glycogen stores are almost depleted after about 1 day of fasting when the levels of insulin and glucagon have adopted a new stable temporal profile. Notably, also for the fasted state, the computed average protein abundance ratios are in good agreement with experimental data which further substantiates the reliability of the model (see Figure 5).

**Figure 8:**
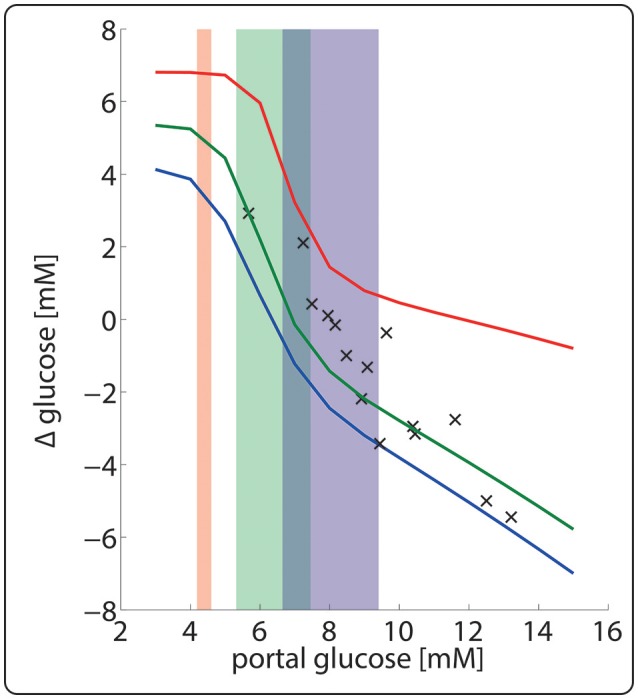
Hepatic porto-venous glucose difference (AVGD) for the well-fed and fasted nutritional state. The daily variations of plasma glucose in the fasted, fed and well-fed state are indicated by the red-, green- and blue-shaded areas. The solid lines represent the AVGD if in the fasted state (red), fed state (green) and the well-fed state (blue). Crosses depict experimentally measured AVGD (Huang and Veech, 1988).

Figure 8 illustrates the importance of dynamic zonation for the adaptation of the porto-venous glucose difference (AVGD) to a specific nutritional regime. Regulation of interconvertible enzymes by hormone-dependent phosphorylation alone, i.e., at fixed protein levels of the fed state (blue line), would result in an AVGD of about 3.5 mM for the typical range of portal glucose concentrations in the fasted state (red shaded area). Dynamic adaption of protein levels enlarges the AVGD to about 7 mM (red line) thus rendering the liver to a strong glucose producer in the fasted state.

### Dynamic Metabolic Zonation of the Liver in Diabetes Type II (“Diabetic Liver”)

**Figure 9:**
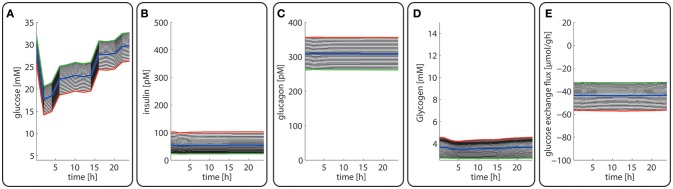
Diurnal variations in the plasma levels of glucose **(A)**, insulin **(B)**, glucagon **(C)**, cellular glycogen **(D)**, and the glucose exchange flux **(E)** in different zones along the porto-central axis of a diabetic rat.

Late diabetes type 2 is characterized by long persistence of high postprandial plasma glucose levels, reduced insulin levels (hypo-insulinemia) and elevated glucagon levels (hyper-glucagonemia). It is mainly the shift in the insulin/glucagon ratio that renders the liver to a glucose producer which on top of the insulin-resistant muscle and adipose tissue contributes to high plasma glucose levels. We tested whether our model can also correctly describe this metabolic abnormality and the observed changes of protein abundances in different zones. To this end, we used the glucose-hormone transfer function constructed for the diabetic case (see Bulik et al., 2016) to calculate the phosphorylation state of interconvertible enzymes and confronted the model over 4 days repeatedly with a 24 h glucose profile of a diabetic rat until an almost stable 24 h pattern of protein abundances had established (Figure 9). As shown in Figure 9D, all hepatocytes work permanently as glucose producers, i.e., at all time points of the day the intra-sinusoidal glucose concentration increases along the portal-central axis (Figure 9A). The glycogen reserves are drastically diminished and the different time courses of glycogen emptying and filling between portal and central regions (see Figure 2D) are completely abolished. Taken together, the zonation of glucose metabolism in the diabetic liver bears a strong resemblance with that of the fasted liver. It is important to note that even in this pathophysiological case the computed average portal-to-central protein abundance ratios are in good agreement with experimental data (see Figure 5).

## Discussion

### Metabolic Zonation of Hepatic Glucose Metabolism Is Driven by Concentration Gradients of Hormones and Metabolites

In this work we used a mathematical model to study the dynamic zonation of the hepatic glucose metabolism. To this end we extended our previously published multi-scale tissue model of the hepatic carbohydrate metabolism (Berndt et al., 2018) by rendering the protein levels of key regulatory enzymes of glycolysis and gluconeogenesis as dynamic model variables which are controlled by timely variable synthesis and degradation in dependence from the concentration of the four modulators glucose, insulin, glucagon and oxygen. The model correctly replicates experimentally determined protein levels in different zones of the liver acinus as well as the adaptation of the liver to a well-fed, fasted and diabetic state. From this we draw four main conclusions. (1) Zonation of the hepatic glucose metabolism is a necessary consequence of the fact that the expression of key regulatory enzymes is controlled by modulators that display a porto-central concentration gradient along the sinusoid. (2) Mechanisms controlling the adaptation of enzyme abundances to varying external conditions necessarily lead to the zonation of hepatic carbohydrate metabolism. (3) The four modulators considered in the model are sufficient to describe the dynamic zonation of the glucose metabolism of the a normal liver. (4) The use of phenomenological transfer functions which directly relate the protein turnover to known modulators of gene expression appears a promising modeling strategy to include variable protein levels in kinetic models in view of the fact that in a foreseeable future explicit kinetic modeling of complex gene-regulatory network is out of reach.

### The Proposed Multi-Scale Model Encompasses All Levels of Metabolic Regulation

An important feature of the cellular metabolic network of the liver is the ability to adapt its functional output to varying external conditions such as changes in nutrient supply and varying hormone levels. These adaptive mechanisms operate at two different time scales. The short term adaptation occurs within seconds or minutes and is brought about by activity changes in the present metabolic enzymes by substrate availability, allosteric regulation and reversible phosphorylation. The second adaptive mechanism operates within hours or days and is brought about by changes in the enzyme abundances. It is already known for a long time that the total protein content of liver enzymes may largely vary owing to enhanced protein degradation during fasting (providing glucogenic amino acids as substrate for gluconeogenesis) mediated by the hormone glucagon or enhanced protein synthesis by the hormone insulin (Hopgood et al., 1980). However, such general changes of the protein content do not tell anything about the changes in the abundance of individual enzymes, whose expression by insulin and glucagon differs profoundly. Therefore, it was necessary to develop empirical rate laws for the synthesis and degradation of individual enzymes. This phenomenological approach was chosen since currently biochemical information is insufficient to establish molecular-resolved kinetic models which include, for example, the interaction of transcription factors among each other and with specific DNA promotor regions, the processing of mRNA and the regulation of mRNA translation by micro RNAs and RNA-binding proteins. For example, PEPCK, probably the best-studied gluconeogenetic enzyme, is regulated by at least a dozen transcription factors with partially unknown interactions (Yang et al., 2009). Even if it was possible to explicitly model the mRNA transcription for individual enzymes, there is still a big gap in understanding post-transcriptional regulation and the processes of post-translational modification.

### Metabolic Response of the Liver to Varying Nutritional Regimes

Our simulations suggest that in the presence of a constant daily nutritional regime the diurnal variation of enzyme abundances should be fairly moderate in the range of 10–20% around the mean. This is a lot less than daily variations in the abundance of the key regulatory enzyme of cholesterol synthesis, ßHMG-CoA reductase (Kirkpatrick et al., 1980), exhibiting a pronounced circadian rhythm or some enzymes of the amino acid metabolism as, for example, tyrosine transaminase the activity of which is almost four times as great several hours after nightfall as it is in the morning (Wurtman, 1974). In contrast, much larger changes of glycolytic and gluconeogenetic enzyme levels are elicited by a switch from well-fed to fasting conditions and vice versa. This metabolic adaptation occurs within a time span of several days (see Figure 6) and enables vertebrates to maintain the plasma glucose level in the absence of food. The slow change of enzyme concentration profiles implies that the capability of the fasted liver to clear a sudden excess of plasma glucose is diminished (impaired glucose tolerance) as the capacity of glycolytic enzymes and enzymes of the glycogen pathway a downregulated (Bulik et al., 2016). Intriguingly, there appears to be a striking similarity in the adaptive response of the liver to fasting conditions and diabetes type 2. In our modeling approach, this is mainly due to the fact that in both physiological settings the strong effect of insulin on the expression of glycolytic and gluconeogenetic enzymes is diminished whereas the effect of glucagon is more pronounced. As demonstrated in a previous model-based simulation study (König and Holzhütter, 2012), exposing the permanently glucose-releasing “starved” liver of the diabetic patient to a rigorous insulin treatment at persistently elevated glucagon level may increase the risk of severe hypoglycaemia. Thus, owing to the equally strong impact of both insulin and glucagon on the expression and phosphorylation state of liver enzymes, reconverting the “starved” liver of the diabetic patient into the normal metabolic phenotype requires the normalization of the plasma levels of both hormones.

### Main Limitations of the Model and Outlook for Future Model Extensions

The used multiscale tissue model comprises a number of simplifications of the true anatomical structure of the liver which may impact on the simulated intra-sinusoidal concentration gradients of hormones and metabolites. For example, the blood flow rate within the pericentral zone of the sinusoid may vary if a sinusoid spreads out, forms anastomoses or merges with another sinusoid (Rappaport et al., 1954), anatomic peculiarities of liver parenchyma that are not yet considered in the STU model. Also, the number of periportal hepatocytes is higher (~2–3-fold) compared to pericentral hepatocytes. Regarding the concentration gradient of oxygen, hormones and metabolites in the sinusoid it may be of relevance that the terminal branches of the hepatic artery rarely join with the portal vein already before the blood enters the sinusoids, as presumed in our model. In the vast majority the merger of arterial blood with blood from the portal vein, occurs a few cells downstream within the sinusoid (Ekataksin and Kaneda, 1999) resulting in a local increase of the concentration of oxygen concentration at this site. Despite these limitations, the model correctly describes glucose exchange rates, gradients and indicator dilution curves for a structurally normal liver (Berndt et al., 2018). Hence, the functional implications of the above limitations and other neglected aspects of the real topology of liver tissue remain unclear. Therefore, in future work we aim to embed our metabolic cell model in a 3-D reconstructions of a mouse lobule (Hoehme et al., 2017).

The rate laws presented in this paper are effective transfer functions describing directly the relation between modulators (nutrients and hormones) and the turnover rate of a protein. Usage of effective transfer function raises the question which properties of the underlying regulatory network have to be captured. Obviously, not all known modulators of protein synthesis and degradation have been considered in the model. Ground-breaking experiments pointed initially to the oxygen gradient as the most important driving force of metabolic zonation (Jungermann and Kietzmann, 1997, 2000). Later experiments with isolated hepatocytes incubated with varying concentrations of insulin or glucagon (Probst et al., 1982) revealed an important role of these hormones for the establishment of liver zonation. Meanwhile a lot more potential modulators of metabolic zonation have been described in the literature, among them Wnt/β-catenin pathway (Torre et al., 2010; Vasilj et al., 2012), MAPK/ERK pathway (Zeller et al., 2013), Hnf4-alpha (Colletti et al., 2009), or thyroid hormones (Weinberg and Utter, 1979). However, as demonstrated in this study, the dynamic zonation of the glucose metabolism can be well described in different physiological settings by taken into account only the four modulators oxygen, glucose, glucagon and insulin. The central role of oxygen, glucose, glucagon and insulin for the dynamic zonation of the glucose metabolism does not exclude that morphogens and growth factors may have an important role in the zonation of other metabolic subsystems (Gebhardt and Matz-Soja, 2014). For example, the expression of the glutamine synthetase is restricted to last few hepatocytes close to the venous pole. Complementary, the urea cycle enzyme carbamoylphosphate synthetase I (CPS I) is present in the periportal, intermediate, and the first few layers of the perivenous zone. It has been clearly demonstrated that Wnt/ß-catenin signaling pathway plays a central role in the stable maintenance of these peculiar zonation profiles (Burke et al., 2009).

## Conclusion

In summary, we propose a self-consistent model of liver carbohydrate metabolism that consistently takes into account variable gene expression of metabolic enzymes, regulation of metabolic pathways, exchange of metabolites and hormones between the blood and hepatocytes and microperfusion of the liver. Once the input of hormones and nutrients to the periportal region the liver acinus is known, the model allows to compute the metabolic phenotype of individual hepatocytes along the porto-central axis. The local hormone and metabolite concentrations determine the phosphorylation state of the interconvertible enzymes, hormonal clearance rates and expression level of metabolic enzymes. The metabolic phenotype in turn determines the functional output (here: glucose exchange rate) of each hepatocyte and this way the venous glucose output of the acinus. Integration across a representative set of acini yields finally the total glucose output of the liver.

## Author Contributions

NB developed the concept, implemented the model, carried out the simulation, wrote the manuscript. H-GH developed the concept, advised the implementation of the model, wrote the manuscript.

### Conflict of Interest Statement

The authors declare that the research was conducted in the absence of any commercial or financial relationships that could be construed as a potential conflict of interest.
