# Supplementary material for: Dynamic Metabolic Zonation of the Hepatic Glucose Metabolism Is Accomplished by Sinusoidal Plasma Gradients of Nutrients and Hormones
Source: Front Physiol. 2018 Dec 12;9:1786. doi: 10.3389/fphys.2018.01786 (PMC6315134; doi:10.3389/fphys.2018.01786)
Supplement: Supplementary file 1 [file Table_1.DOCX]

Supplement: Validation

Glucose Transporter

Synthesis rate of glucose transporter in dependence of glucose. Experimental data taken from [[1](#_ENREF_1)]


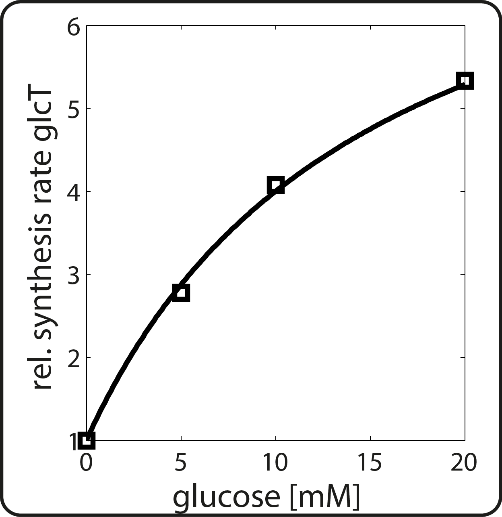


Glucokinase

Synthesis rate of glucokinase in dependence of insulin and oxygen. Experimental data taken from [[2](#_ENREF_2)],[[3](#_ENREF_3)]


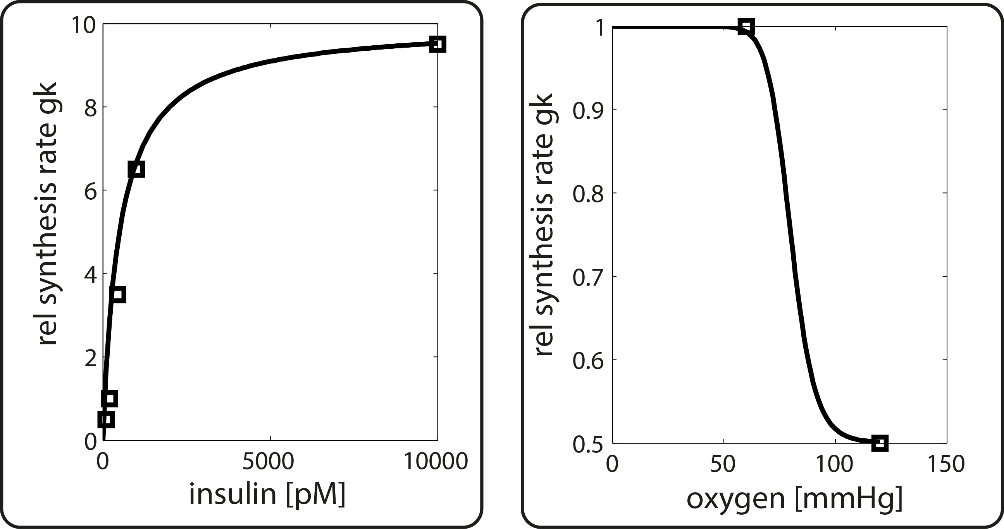


Glucose-6-pohosphatase

Synthesis rate of Glucose-6-pohosphatase mRNA in dependence of glucose and synthesis rate of Glucose-6-pohosphatase in dependence glucagon. Experimental data taken from [[4](#_ENREF_4), [5](#_ENREF_5)]. An translation efficiency of 30 % [[4](#_ENREF_4)] was taken into account (see factor of 0.3 in Table 1).


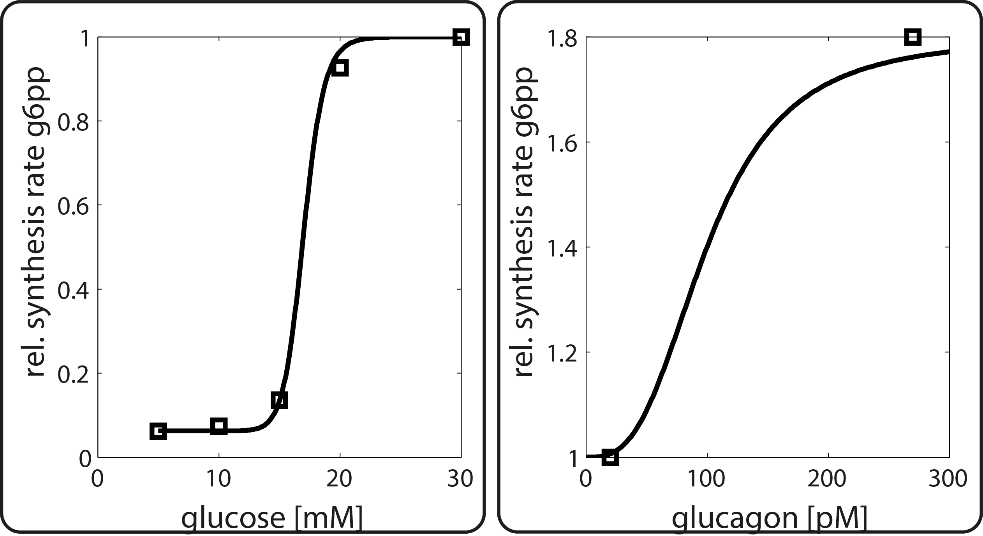


PFK1

Degradation rate of phosphofructokinase1 in dependence of insulin. Experimental data taken from [[6](#_ENREF_6)].


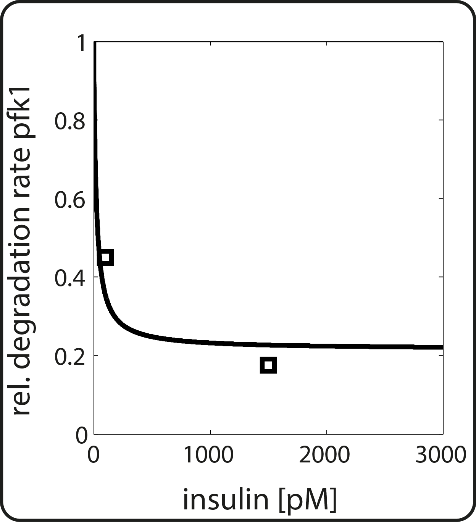


FBP1

Synthesis rate of fructose-1,6 phosphatase in dependence of glucagon. Experimental data taken from [[7](#_ENREF_7)]. Since cAMP is generated by glucagon dependent activation of adenylate cyclase maximal cAMP levels were assumed at saturating glucagon levels of 10 nM.


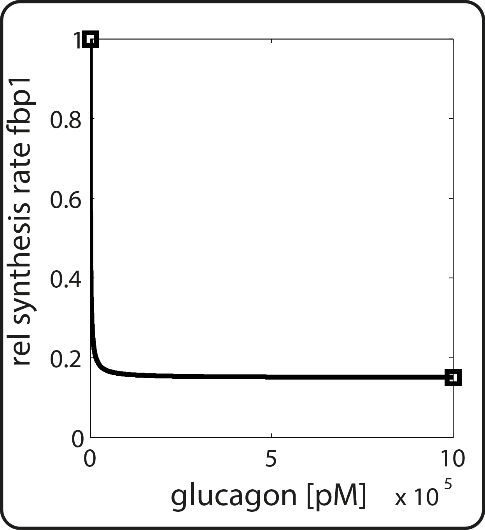


PFK2/FBP2

Synthesis rate of the tandem enzyme phosphofructokinase 2 /frucotse2,6 phosphatase in dependence of oxygen and glucagon. Experimental data taken from [[8](#_ENREF_8), [9](#_ENREF_9)].


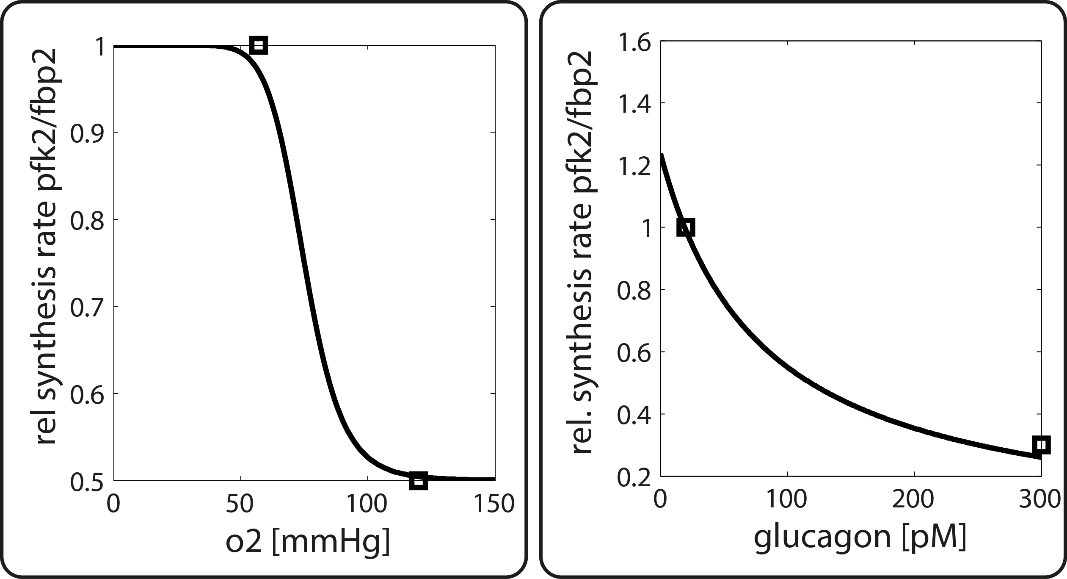


Pyruvate Kinase

Synthesis rate of the pyruvate kinase in dependence of oxygen. Relative synthesis rate of pyruvate kinase in the normal and diabetic case. Normal case was simulated by 1000 pM insulin and 20 pM glucagon, diabetes was simulated by 100 pM insulin and 300 pM glucagon (see [[10](#_ENREF_10)]). Black bars show simulation results, red bars depict the experimental values. Experimental data taken from [[11](#_ENREF_11), [12](#_ENREF_12)]


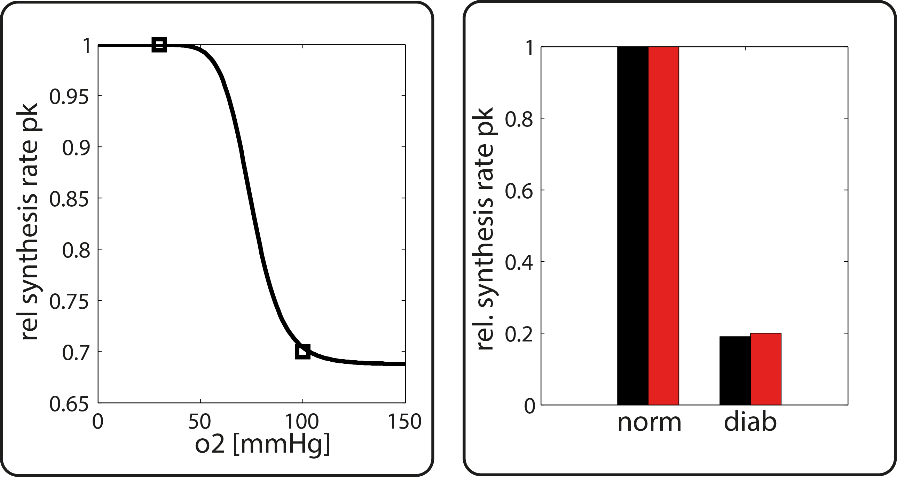


Pyruvate Carboxylase

Relative synthesis rate of pyruvate carboxylase in the normal and diabetic case. Normal case was simulated by 1000 pM insulin and 20 pM glucagon, diabetes was simulated by 100 pM insulin and 300 pM glucagon ([[10](#_ENREF_10)]. Black bars show simulation results, red bars depict the experimental values, error bar depicts variance in experimental values. Experimental data taken from [[13](#_ENREF_13)] .


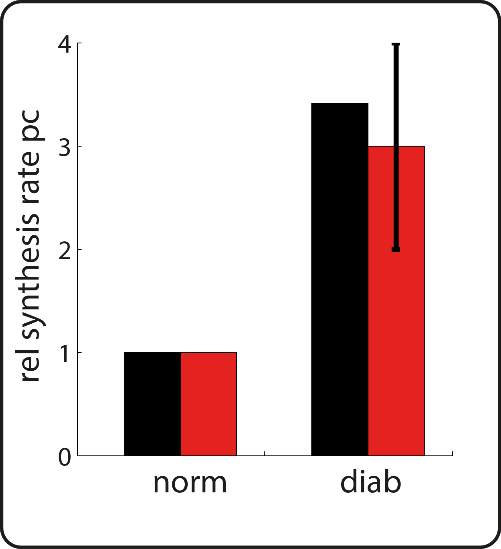


Phosphoenolpyruvatec arboxykinase

Synthesis rate of the phosphoenolpyruvate carboxykinase in dependence of oxygen, insulin and glucaogon. The optimal fit for the oxygen dependency (red curve) gives a $k_{2}=0.7$ . Instead $k_{2}=2$ was used (black curve) to match observed pp-pc ratios. Experimental data taken from [[14](#_ENREF_14), [15](#_ENREF_15)]


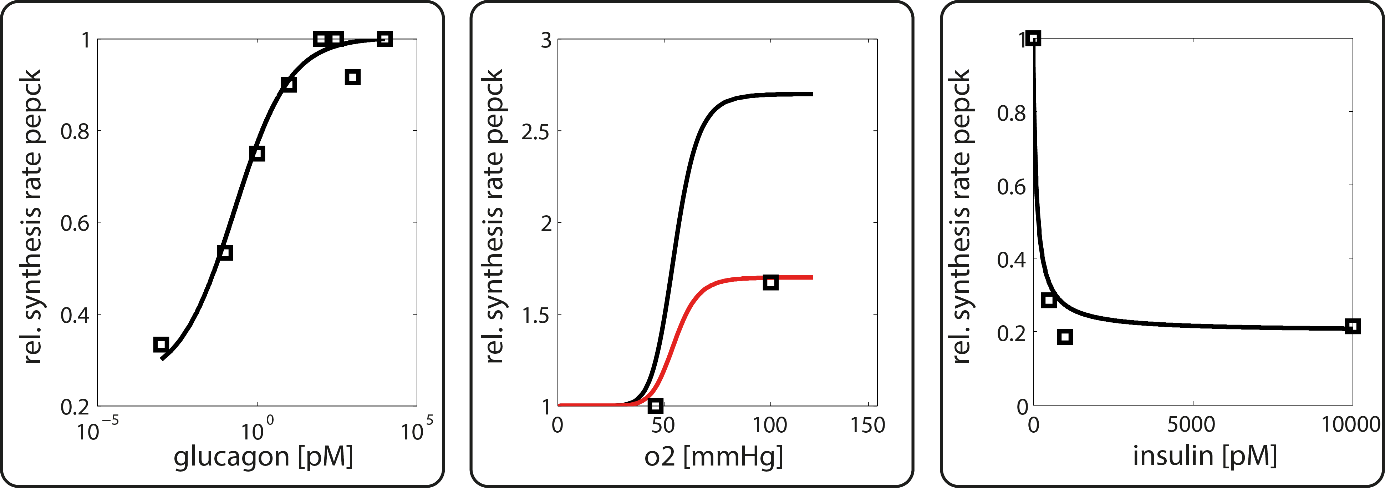


1. Postic, C., et al., *Evidence for a transient inhibitory effect of insulin on GLUT2 expression in the liver: studies in vivo and in vitro.* Biochem J, 1993. **293 ( Pt 1)**: p. 119-24.

2. Iynedjian, P.B., et al., *Transcriptional induction of glucokinase gene by insulin in cultured liver cells and its repression by the glucagon-cAMP system.* J Biol Chem, 1989. **264**(36): p. 21824-9.

3. Kietzmann, T., et al., *Arterial oxygen partial pressures reduce the insulin-dependent induction of the perivenously located glucokinase in rat hepatocyte cultures: mimicry of arterial oxygen pressures by H2O2.* Biochem J, 1997. **321 ( Pt 1)**: p. 17-20.

4. Massillon, D., *Regulation of the glucose-6-phosphatase gene by glucose occurs by transcriptional and post-transcriptional mechanisms - Differential effect of glucose and xylitol.* Journal of Biological Chemistry, 2001. **276**(6): p. 4055-4062.

5. Argaud, D., et al., *Regulation of rat liver glucose-6-phosphatase gene expression in different nutritional and hormonal states: gene structure and 5'-flanking sequence.* Diabetes, 1996. **45**(11): p. 1563-71.

6. Dunaway, G.A., Jr. and G. Weber, *Effects of hormonal and nutritional changes on rates of synthesis and degradation of hepatic phosphofructokinase isozymes.* Arch Biochem Biophys, 1974. **162**(2): p. 629-37.

7. Elmaghrabi, M.R., et al., *The Rat Fructose-1,6-Bisphosphatase Gene - Structure and Regulation of Expression.* Journal of Biological Chemistry, 1991. **266**(4): p. 2115-2120.

8. Minchenko, O., I. Opentanova, and J. Caro, *Hypoxic regulation of the 6-phosphofructo-2-kinase/fructose-2,6-bisphosphatase gene family (PFKFB-1-4) expression in vivo.* Febs Letters, 2003. **554**(3): p. 264-270.

9. Rosa, J.L., et al., *Regulation of Hepatic 6-Phosphofructo-2-Kinase Fructose 2,6-Bisphosphatase Gene-Expression by Glucagon.* Journal of Biological Chemistry, 1993. **268**(30): p. 22540-22545.

10. Bulik, S., H.G. Holzhutter, and N. Berndt, *The relative importance of kinetic mechanisms and variable enzyme abundances for the regulation of hepatic glucose metabolism - insights from mathematical modeling.* Bmc Biology, 2016. **14**.

11. Wolfle, D. and K. Jungermann, *Long-Term Effects of Physiological Oxygen Concentrations on Glycolysis and Gluconeogenesis in Hepatocyte Cultures.* European Journal of Biochemistry, 1985. **151**(2): p. 299-303.

12. Noguchi, T., H. Inoue, and T. Tanaka, *Transcriptional and Post-Transcriptional Regulation of L-Type Pyruvate-Kinase in Diabetic Rat-Liver by Insulin and Dietary Fructose.* Journal of Biological Chemistry, 1985. **260**(26): p. 4393-4397.

13. Weinberg, M.B. and M.F. Utter, *Effect of streptozotocin-induced diabetes mellitus on the turnover of rat liver pyruvate carboxylase and pyruvate dehydrogenase.* Biochem J, 1980. **188**(3): p. 601-8.

14. Gabbay, R.A., et al., *Insulin regulation of phosphoenolpyruvate carboxykinase gene expression does not require activation of the Ras/mitogen-activated protein kinase signaling pathway.* J Biol Chem, 1996. **271**(4): p. 1890-7.

15. Nauck, M., et al., *Modulation of the Glucagon-Dependent Induction of Phosphoenolpyruvate Carboxykinase and Tyrosine Aminotransferase by Arterial and Venous Oxygen Concentrations in Hepatocyte Cultures.* European Journal of Biochemistry, 1981. **119**(3): p. 657-661.
